# Skeletal muscle mechanics: questions, problems and possible solutions

**DOI:** 10.1186/s12984-017-0310-6

**Published:** 2017-09-16

**Authors:** Walter Herzog

**Affiliations:** Faculty of Kinesiology, University of Calgary, 2500 University Dr, Calgary, AB T2N-1N4 Canada

**Keywords:** Muscle mechanics, Cross-bridge Theory, Sarcomeres, Residual Force Enhancement, Muscle Modeling, Force Sharing, Sliding Filament, Titin

## Abstract

Skeletal muscle mechanics have been studied ever since people have shown an interest in human movement. However, our understanding of muscle contraction and muscle mechanical properties has changed fundamentally with the discovery of the sliding filament theory in 1954 and associated cross-bridge theory in 1957. Nevertheless, experimental evidence suggests that our knowledge of the mechanisms of contraction is far from complete, and muscle properties and muscle function in human movement remain largely unknown.

In this manuscript, I am trying to identify some of the crucial challenges we are faced with in muscle mechanics, offer possible solutions to questions, and identify problems that might be worthwhile exploring in the future. Since it is impossible to tackle all (worthwhile) problems in a single manuscript, I identified three problems that are controversial, important, and close to my heart. They may be identified as follows: (i) mechanisms of muscle contraction, (ii) in vivo whole muscle mechanics and properties, and (iii) force-sharing among synergistic muscles. These topics are fundamental to our understanding of human movement and movement control, and they contain a series of unknowns and challenges to be explored in the future.

It is my hope that this paper may serve as an inspiration for some, may challenge current beliefs in selected areas, tackle important problems in the area of muscle mechanics, physiology and movement control, and may guide and focus some of the thinking of future muscle mechanics research.

## Background

On June 12–16, 2016, approximately 150 scientists in the areas of biomechanics and neural control of movement met at the Deer Creek Lodge in Sterling Ohio for an unusual meeting. The meeting was unusual since it only had happened once before, 20 years earlier, and it was unusual because half of the available time was set aside for discussion, thus the ratio of discussion time vs. presentation time was highly favorable for those who like to discuss things.

I was invited to this conference with the mandate to chair a session on skeletal muscle mechanics, energetics and plasticity. The task given to me was to identify some of the major questions and problems in skeletal muscle mechanics and present those in a concise manner and understandable to the non-expert. I must admit this was a rather difficult task for a person like me who believes that we know little to nothing about muscle contraction (on the molecular level), what the basic muscle properties are (except for the most standardized conditions), and how muscles function in the in vivo, freely moving system under non-steady-state, submaximal conditions. In the end, I identified three topics that I presented and discussed. These topics, in my opinion, comprise some of the most relevant questions in muscle mechanics and movement control, but they do not comprise, by any means, the full set of questions/problems in this area of research.

At the end, I settled on topics that are highly controversial, often misunderstood, and close to my heart. They may be summarized as follows: (i) Mechanisms of muscle contraction, sarcomere stability and mechanics, (ii) whole muscle mechanics and muscle properties, and (iii) force-sharing among synergistic muscles. In the following, I will be discussing these topics concisely by raising one or more problems in the area, provide possible solutions, and may make some suggestions for future challenges that, if solved, may improve our understanding of skeletal muscle biomechanics and movement control.

Following my introductory manuscript will be four manuscripts supplied by the participants of the muscle workshop: Drs. Rick Lieber, Tom Roberts, Silvia Blemker and Sabrina Lee. Their contributions are focused on specific problems and challenges faced today by researchers in muscle mechanics and they will add important considerations to the discussion below. I sincerely hope that the BANCOM conference will be repeated in another twenty years, and that we can reflect on which of the challenges, questions and problems have been solved. Hopefully, the set of papers presented here will form a framework for what some of the young people entering this field may consider worthwhile projects.

### Mechanisms of muscle contraction, sarcomere stability and mechanics

#### The cross-bridge theory (description)

When opening a textbook of muscle physiology and searching for how muscles contract, we are inevitably exposed to the cross-bridge theory of contraction. This theory was first proposed in a rather obscure journal (Progress in Biophysics and Biophysical Chemistry) that only existed for a brief period of time. The founding editor of that journal was a friend of Andrew Huxley, and so he asked his friend to make a contribution, and Huxley [1] submitted his ideas of how muscles may contract. Andrew Huxley confided in me that he never wanted this paper to be published, that he thought it was too preliminary and needed more refinement, and if it was not for his friend, he would never have considered sending such a preliminary report to any journal (Huxley-Herzog discussion August, 1999). This “preliminary” report that was never meant for public presentation has gathered 3428 citations (as of Dec, 16, 2016).

**Fig. 1:**
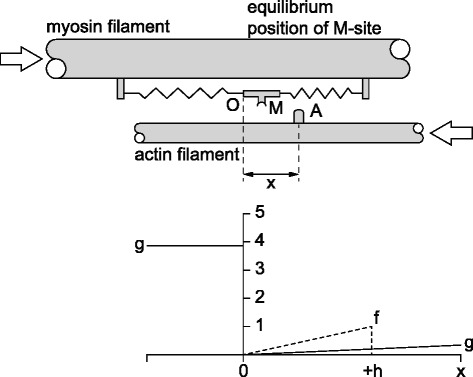
Schematic representation of the original cross-bridge model with a myosin cross-bridge cyclically interacting with specific attachment sites on the actin filament. In the lower part of the figure is a representative illustration of the asymmetrical rate constants of attachment (f) and detachment (g) that are thought to govern the cross-bridge kinetics. Also shown is the so-called “x-distance” on the top and bottom parts of the figure, which is defined as the distance from the cross-bridge equilibrium position to the nearest eligible attachment site on actin. (Adapted from Huxley [1], with permission)

The cross-bridge theory states that contraction and force production in muscles is caused by the cyclic interaction of side-pieces (cross-bridges) originating from myosin filaments with actin filaments (Fig. 1). The cross-bridges are thought to be attached to the myosin filaments via an elastic link, and cross-bridges are moved by Brownian motion from the equilibrium position of this elastic link to positions where the elastic link bears substantial forces (2-4pN). Interaction of these cross-bridges with the actin filaments was then thought to be governed by rate constants of cross-bridge attachment and detachment that were exclusively dependent on Huxley’s so-called x-distance (Fig. 1): the distance from the cross-bridge equilibrium position to the nearest eligible attachment site on actin.

The cross-bridge theory of muscle contraction was based on some fundamental assumptions that included the following:

(i) Cross-bridges are uniformly arranged along the myosin filaments
(ii) Cross-bridge attachment sites on actin are uniformly arranged along the actin filament
(iii) Each cross-bridge has the same force potential
(iv) Cross-bridge force is exclusively governed by the elongation of the (linearly) elastic link that connects cross-bridges to the myosin filament backbone
(v) Cross-bridges are independent of each other
(vi) Cross-bridge attachment and detachment is determined by rate constants that depend exclusively on the “x-distance” (Fig. 1) and
(vii) Each cross-bridge cycle is associated with the hydrolysis of one high energy phosphate compound – ATP (adenosine triphosphate)

**Fig. 2:**
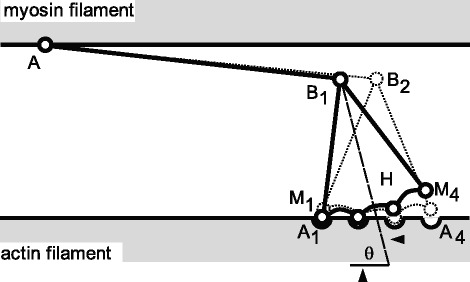
Refinement of the original (1957) cross-bridge theory by assuming that cross-bridge force production occurs through a rotation (rather than a linear translation) of cross-bridges, and further assuming that cross-bridge attachment has multiple (rather than a single) states. (Adapted from Huxley and Simmons [3], with permission)

Refinements of the cross-bridge theory were made by including a rotating cross-bridge motion (rather than just the linear cross-bridge motion of the initial theory – [2, 3], a multi-state attached and detached cross-bridge model [3] (Fig. 2), and a detailed atomic description of the structure of cross-bridges and corresponding attachment sites on actin [4].

### The cross-bridge theory (problems)

The cross-bridge theory captures many experimental properties of muscles well, and there is little doubt that actin-myosin interactions through cross-bridges are an important and integral part of muscle contraction mechanisms and force production. The cross-bridge theory gives a ready explanation for some of the mechanical properties of skeletal muscles, such as the force-length relationship [5]. Specifically, the so-called descending limb of the force-length relationship is well explained with the decrease in overlap between actin and myosin filaments as sarcomere lengths go beyond those at which maximal active force can be produced. The cross-bridge theory can also be adapted (by proper choice of the rate functions for attachment and detachment) to predict the force-velocity relationship [6] of shortening muscle well.

However, from its very beginnings, the cross-bridge theory had difficulty predicting forces, energetics, and stiffness of muscles in eccentric (actively lengthening) contractions properly [1, 7]. The cross-bridge theory also cannot predict the history-dependent properties, such as residual force enhancement [8], and residual force depression [9] without substantial changes to the fundamental assumptions of the theory [10]. Finally, the cross-bridge theory also predicts instabilities of half-sarcomere and sarcomere forces and lengths on the descending limb of the force-length relationship [11–13], thereby rendering approximately 60% of the working range of a muscle useless, a prediction that turns out to be not correct.

Fortunately, these shortcomings of the cross-bridge theory can all be eliminated in a straight forward manner, with a single assumption, and a simple addition to the cross-bridge theory that leaves the cross-bridge theory fully intact [14–17]. This addition includes a spring element connecting the actin and myosin filaments, and the assumption that this spring element has a variable stiffness, with stiffness increasing with activation and/or active force production. Let me illustrate two selected problems of the cross-bridge theory in more detail: (i) residual force enhancement and (ii) sarcomere force/length instability.

#### Residual force enhancement

**Fig. 3:**
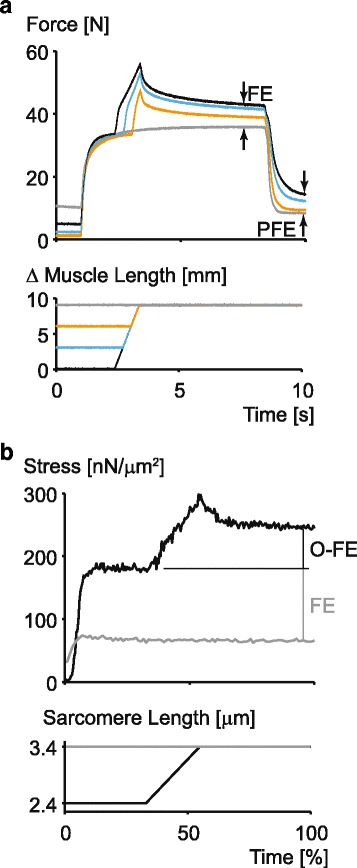
Force enhancement property of skeletal muscle as experimentally observed in a whole, intact muscle **a** and in a single, mechanically isolated sarcomere **b**. Note that the steady-state isometric force following an active stretch is substantially greater than the corresponding steady-state force for a purely isometric reference contraction at the same length and with the same amount of activation (indicated as FE in both figures). Furthermore, the force enhancement often also contains a passive component, indicated by PFE in fig. (**a**). Note also, the increase in force above that observed at optimal sarcomere length following active stretching of a single sarcomere (O-FE in Fig. **b**). Finally, note that the amount of force enhancement is increased with increasing stretch magnitude (in Fig. **a**)

When an active muscle is stretched (eccentric contraction), its steady-state isometric force following the stretch is greater than the corresponding (same length, same activation) steady-state, isometric force for a purely isometric contraction (e.g. [8] (Fig. 3). We demonstrated that this residual force enhancement was caused, at least in part, by a passive structural element [18] (see also the passive force enhancement PFE in Fig. 3a). However, the cross-bridge theory predicts that steady-state forces depend only on the length and the speed of contraction of the muscle, and when these are identical (i.e. in our case – same length and isometric – zero velocity – contraction) then the forces are predicted to be identical. However, this is not the case. Residual force enhancement has been demonstrated to occur on all structural levels of muscle ranging from measurements on single, mechanically isolated sarcomeres [19] to fully intact, voluntarily activated human skeletal muscles (e.g. [20]).

Problem: the cross-bridge theory cannot predict history-dependent properties in general and residual force enhancement properties specifically, despite overwhelming experimental evidence and general acceptance in the scientific community that these properties exist on all structural levels of muscle.

#### Sarcomere and half-sarcomere length (in)stability

**Fig. 4:**
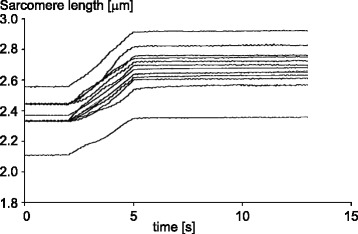
Representative sarcomere length traces as a function of time for all individual sarcomeres of a single myofibril. The myofibril in this experiment was actively stretched from an initial average sarcomere length on the plateau of the force-length relationship to a final length on the descending limb of the force-length relationship. Note that the individual sarcomeres are at vastly different lengths that are associated with active force differences of up to 100%, but the sarcomere lengths are perfectly stable (constant) despite these predicted force differences. The cross-bridge theory, as well as the sarcomere instability theory predict that the longest (weakest) sarcomeres are pulled quickly beyond actin myosin filament overlap (lengths greater than 3.9 μm in this preparation), at the expense of the shortest (strongest) sarcomeres, but this clearly does not happen. Therefore, there must be stabilizing elements in single, serially arranged sarcomeres in a myofibril that have not been considered in the cross-bridge theory

In the cross-bridge theory, force is exclusively produced by the interaction of actin and myosin filaments. Since interactions of actin and myosin occur in a stochastic way, the number of cross-bridges attached in the left half and right half of a sarcomere differ in general. If one half sarcomere has more cross-bridges attached than the other, it produces more force and thus will be shortening at the expense of the other half. On the descending limb of the force-length relationship, this will result in an increased actin-myosin filament overlap zone in the half sarcomere that has shortened and less overlap in the half sarcomere that was elongated. This situation will result in an increased probability of cross-bridge attachment for the short half sarcomere compared to the long half sarcomere, thereby making the force difference between the two half sarcomeres greater. This produces an unstable situation where one half sarcomere will end up shortened (i.e., the myosin – A-band – is pulled to one side of the sarcomere) while the other half sarcomere is left with little or no actin-myosin filament overlap. A similar argument for instability on the descending limb of the force-length relationship has been made for entire muscle segments [21], and for single sarcomeres [22]. However, when stretching sarcomeres in a single myofibril to lengths on the descending limb of the force-length relationship, all sarcomeres undergo a (variable) stretch and remain at constant, but vastly different, (half-) sarcomere lengths after stretch, thereby demonstrating perfectly stable properties [23, 24] (Fig. 4).

Problem: The cross-bridge theory predicts inherent instabilities in sarcomere and half sarcomere lengths on the descending limb of the force-length relationship, while experimentally such instabilities are not observed.

### The cross-bridge theory (possible solutions)

In the two-filament model of the cross-bridge theory, actin and myosin are the lone active force producing elements and their interaction is based on stochastic events. In order to produce half-sarcomere and sarcomere stability independent of sarcomere lengths, account for the experimentally observed residual force enhancement, and explain experimentally observed inconsistencies in the energetics and force trajectories in eccentric muscle contraction, a structural element connecting myosin with actin would be an elegant solution. If this structural element had spring-like properties, and could adjust its spring stiffness in an activation/force-dependent manner, then all of the experimental observations of eccentric muscle contraction (sarcomere stability, force enhancement, energetic savings) could be explained in a simple and straight forward manner.

The structural protein titin (also called connectin) was discovered in the mid- to late-1970s [25, 26], and it satisfies the above criteria. It runs across the half sarcomere inserting in the M-band of the sarcomere and connects (firmly) to the myosin filaments distally and actin filaments and the Z-line proximally. In the I-band region, titin runs freely and elongates against resistance, and shortens when resistance is removed. Therefore, titin is often referred to as a molecular spring that is virtually elastic prior to the unfolding of its immunoglobulin (Ig) domains, but becomes highly viscous once the Ig domains are being unfolded. However, unfolding of Ig domains is thought to occur primarily at lengths greater than the normal physiological range of muscles in situ [27, 28].

Over the past twenty years, it has been discovered that titin can change its spring stiffness in a variety of ways, for example by binding calcium and by phosphorylation of specific titin sites. Calcium binding to the glutamate rich region of titin’s PEVK segment and to selected cardiac Ig domains upon muscle activation has resulted in increases in titin stiffness and force upon stretch [29, 30].

**Fig. 5:**
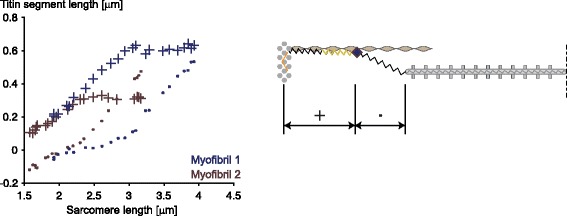
Proximal (designated with cross signs) and distal titin segment lengths (dots) in single sarcomeres of a myofibril stretched while it is in an activated state. Note that the proximal and distal titin segments initially elongate linearly with the elongation of the sarcomere, but after a short stretch, the proximal segment stops elongating while the distal segment accommodates the entire sarcomere stretch. We interpret this result as an attachment of the proximal titin segment to actin after a short stretch distance, thereby only leaving the short and stiff distal segment to accommodate the sarcomere elongation. If correct, this binding of titin to actin (predicted theoretically to occur in the middle of the so-called PEVK segment of titin [33]) would increase titin’s stiffness dramatically, thereby causing increased titin forces in actively compared to passively stretched sarcomeres. When myofibrils are stretched passively, the proximal and distal segments are stretched throughout the entire stretch phase in the same manner as indicated in this figure prior to titin attachment to actin, indicating that titin to actin binding does not take place in passively stretched muscles (results not shown)

**Fig. 6:**
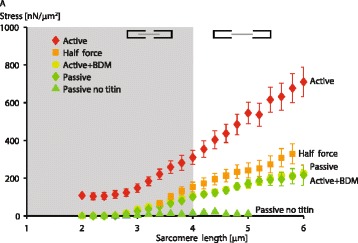
Stress vs. average sarcomere length traces for experiments in single myofibrils stretched way beyond actin-myosin filament overlap while activated (Active), while passive (Passive), and after elimination of titin (Passive no titin). In the region beyond actin-myosin filament overlap (beyond the grey shaded area), one would expect the force in the passively and actively stretched sarcomeres to be the same as cross-bridge based active forces are eliminated in this region. However, this was not the case and sarcomeres stretched beyond actin-myosin filament overlap had titin-based forces that were 3–4 times greater in actively compared to passively stretched myofibrils when stretching started at a sarcomere length of 2.0 μm. When stretching started at average sarcomere length of 3.4 μm (that is halfway down the descending limb of the force-length relationship – Half force), the extra, titin-based force, was substantially reduced but still significantly greater than the corresponding forces obtained in passive stretching of myofibrils. When titin is eliminated from the myofibril preparation, all passive and active force production is eliminated as well, indicating that (i) titin is required for active force transmission, and (ii) that titin is the only force carrying structure in single sarcomeres once sarcomeres are stretched beyond actin-myosin filament overlap. Combined, these results suggest that titin produces more force in actively compared to passively stretched muscles. The mechanisms of how this titin-based increases in force are achieved remain unknown but are thought to occur through an increase in titin stiffness caused by calcium binding to titin upon activation as shown by Labeit and Duvall [29, 30], and by titin binding to actin as shown in our laboratory [16, 17]. (Adapted from Herzog and Leonard [31], with permission)

**Fig. 7:**
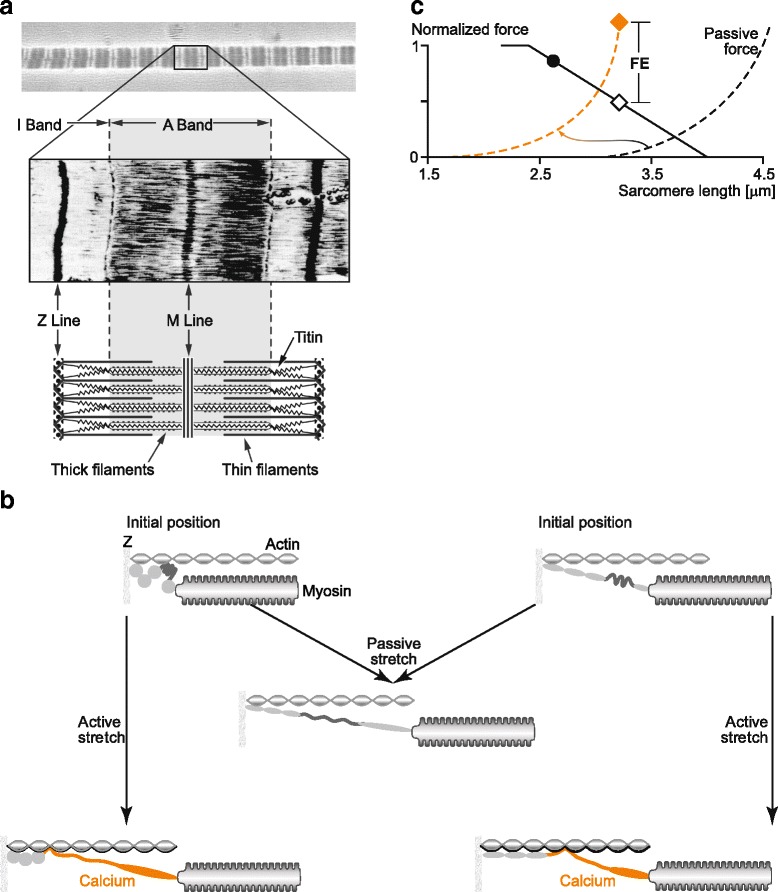
Proposed mechanism of force production in skeletal muscles including the “activation” of titin and its variable contribution to force production in skeletal muscles vis a vis the cross-bridge based actin-myosin based active forces. **a** Micrographs of serially arranged sarcomeres and a single sarcomere, plus schematic representation of a single sarcomere containing titin as the third filament besides actin and myosin. **b** Proposed mechanism of titin-based increase in force upon activation. Upon muscle activation, titin is thought to bind calcium, thereby increasing its inherent spring stiffness, and also to bind its proximal segment to actin, thereby shortening its free spring length and thus further increasing its stiffness. The left and right top figures indicate two different initial sarcomere lengths. Stretching the sarcomere passively to a given length will lead to the same passive force (centre) and titin is stretched without attaching to actin. Stretching the sarcomere actively to a given length (left and right bottom figures) will result in increased titin-based force because of calcium binding to titin and titin binding to actin, as explained in the text. Forces in the actively stretched sarcomere will depend on the initial length prior to the start of stretching, because titin is thought to attach at different points on actin, predicting that a longer stretch distance (bottom left figure) will result in a more increased force than a shorter stretch distance (bottom right figure). **c** Schematic illustration of the change in passive (titin-based) force between passive and active stretches of skeletal muscles. In the active stretch, the passive force starts at a shorter sarcomere (muscle) length, and passive force is stiffer than for the passive stretch because of the engagement of titin with actin and because of calcium binding to titin upon muscle activation. Note, how far the shift in passive force is, and how much stiffer the passive (titin-based) force is in actively compared to passively stretched muscle depends crucially on the initial sarcomere length and the amount of stretch. (Adapted from Herzog [14], with permission)

Recently, there has also been evidence that proximal segments of titin might bind to actin in the presence of activation and active force production, thereby shortening its spring length, increasing its stiffness, and thus force, upon stretching [16, 17] (Fig. 5). Evidence from single sarcomeres and myofibrils pulled to sarcomere lengths way beyond actin-myosin filament overlap while activated were associated with an increase in titin stiffness and force of up to 3–4 times of that observed by passive elongation [31, 32] (Fig. 6). These findings are strong evidence that titin stiffness and force are regulated by activation and active force production, thereby providing a simple explanation for many observations that remain unexplained with the 2-filament sarcomere model of the cross-bridge theory. These hitherto unexplained phenomena include the residual force enhancement, sarcomere and half-sarcomere stability, and the low energetic cost of eccentric contraction, which are readily explained with a 3-filament sarcomere model that includes titin as an activatable spring whose stiffness can be modulated by muscle activation and actin-myosin-based force production [33] (Fig. 7).

Briefly, residual force enhancement in a 3-filament sarcomere (including titin) can be explained with the engagement of titin with actin and/or the stiffening of titin when a muscle is activated [14, 33–38]. Titin binding to actin upon activation is thought to decrease the free spring length of titin and therefore make it stiffer [15]. A stiffer titin would then produce more force when a muscle is stretched actively compared to when the muscle is stretched passively. The same is true for titin stiffening upon activation. It has been shown that in active muscle, calcium binds to specific sites on titin (e.g. the glutamate rich region of the so-called PEVK domain [29, 39], and selected immunoglobulin (Ig) domains [30], thereby increasing titin’s stiffness and force upon active stretching compared to passive stretching. Therefore, the residual force enhancement can be explained by the engagement of titin upon activation as has been suggested based on early theoretical [35, 37], and first ever experimental evidence of passive contributions to the force enhancement property of skeletal muscle [18]. In summary, there is good evidence that titin force is greater when a muscle is actively stretched compared to when it is passively stretched, and this additional force can explain at least part of the residual force enhancement property.

Sarcomere and half-sarcomere stability can be explained by titin, because titin has been shown to centre the myosin filament [40, 41]. In the absence of titin, neither passive nor active forces can be transmitted from one end of a sarcomere to the other end, sarcomeres and half-sarcomeres become unstable and no force can be produced [31]. Titin provides stability to the half-sarcomere by providing resistance when thick filaments are moved away from the centre of the sarcomere. In active muscle, when titin’s stiffness is known to be increased, titin provides a potential energy well for the thick filaments, thus providing stability. Similarly, when sarcomeres and single myofibrils are stretched in an activated preparation, force will continuously increase because of the increased stiffness in titin in active compared to passive muscle, thus providing positive stiffness at all lengths, including the descending limb of the force-length relationship and even when sarcomeres are pulled beyond actin-myosin filament overlap. This positive stiffness provides the stability to half- and full sarcomeres on the descending limb of the force-length relationship, as first shown by us when pulling single myofibrils onto the descending limb of the force-length relationship and observing perfect sarcomere length stability in the presence of great sarcomere length non-uniformities [23].

Finally, the reduced metabolic cost of eccentric contractions, and the reduced ATP consumption per unit of force for muscles in the force-enhanced compared to a purely isometric reference state [42] can also be explained with titin. According to the titin contraction theory [14, 15, 17, 36], titin binds to actin upon muscle activation and stays bound even when the muscle is deactivated [18]. Binding of titin comes at virtually no metabolic cost, and titin’s additional force in eccentric contraction comes at zero cost, thereby reducing the energetic cost of eccentric contractions compared to that of concentric and isometric contractions where all force essentially comes from actin-myosin based cross-bridge interactions which cost one ATP per cross-bridge cycle. Replacing some of the eccentric force with a structural element, such as titin, thus reduces the metabolic cost of eccentric contractions and makes them energetically highly efficient.

### The cross-bridge theory (future challenges)

The fact that the cross-bridge theory on its own produces muscle force and sarcomere length instabilities [5, 21, 22, 43], cannot account for residual force enhancement and other time-dependent properties of muscles [8, 9, 44], and is unable to predict the energetics and force changes in eccentric contractions properly [1, 7] has been known for a long time. However, powerful and unreserved support for the cross-bridge theory, and its beautiful predictive properties for steady-state isometric and concentric conditions, has resulted in a diminished attention to the shortcomings of this theory. Even to date, many scientists believe that sarcomeres are unstable on the descending limb of the force-length relationship and that residual force enhancement and other time-dependent properties can be accounted for by assuming that selected sarcomeres are rapidly pulled beyond actin-myosin filament overlap (they are thought to pop), despite ample direct evidence to the contrary.

Therefore, the future challenges relating to the molecular mechanisms of muscle contraction may be summarized as follows:

1. Determine the role of non-actin myosin-based force regulation. Specifically, determine how titin’s stiffness is modulated upon activation and force production. Although it is known that calcium binding and phosphorylation affect titin’s stiffness, how and where this occurs in detail remains unexplained.
2. Titin is thought (by some) to bind to actin, thereby shortening its spring stiffness and force upon muscle (sarcomere) stretching. Determine if this is indeed correct, and identify the possible binding sites between titin and actin and what forces these binding sites can withstand. In conjunction with this work, and if titin indeed binds to actin, then it becomes likely that Ig domain unfolding will occur at physiologically relevant muscle length. The kinetics of Ig domain unfolding and refolding will then become a crucial aspect of force production in muscle and needs to be determined in great detail.
3. Identify if there are structural proteins other than titin that might be involved in muscle force regulation.
4. Identify if sarcomeres are indeed the smallest independent contractile units in muscle. Evidence suggests that serially arranged sarcomeres in a myofibril are not independent of each other. Rather it appears that force along sarcomeres is collectively controlled, either by mechanical connections between sarcomeres or by feedback systems that regulate cross-bridge kinetics. The former solution is more appealing as it merely requires cross-connections across the Z-band, while the latter would require a sensing and information exchange mechanism between serially arranged sarcomeres in a myofibril.

### Whole muscle mechanics and properties

Similar to our restricted understanding of how muscles contract on the molecular level, there is much to learn about in vivo muscle function. The basic properties associated with muscle force production are the force-length relationship [5], the force-velocity relationship [6] and the history (or time)-dependent properties of residual force enhancement and force depression [44]. Even though these properties represent the basis for all muscle function, we know virtually nothing about them for in vivo muscle contraction. For example, I could ask the question, what is the force-length, force-velocity, and history-dependent property of the human rectus femoris muscle, and nobody would be able to give a satisfactory answer. For the purpose of analysis, let’s focus on arguably the simplest, most recognized, and most discussed property of human skeletal muscles: the force-length relationship.

#### The force-length relationship (problems)

The force-length relationship describes the relationship between the maximum, active, steady-state isometric force of a muscle and its lengths, where lengths may be represented by the entire muscle tendon unit, a fascicle/fibre, or even a single sarcomere [45]. Typically, for human muscle function, researchers rely on the moment-angle relationship of a muscle, rather than the force-length relationship. This representation has many advantages. For example, human joint moments can be readily measured using specialized and commercially available dynamometers, and joint angles can be determined with great accuracy while muscle lengths cannot. Nevertheless, moment-angle relationships typically represent the moments produced by a synergistic group of muscles, and often are thought to contain antagonistic contributions. Therefore, if we want to know the contribution of a single muscle to the resultant joint moment, basic and non-trivial assumptions need to be made. For example, when measuring maximal isometric knee extensor moments, the contribution of a single muscle (let’s say the vastus lateralis) is often calculated based on its relative cross-sectional area [46]. So, if the relative physiological cross-sectional area of the vastus lateralis relative to the entire knee extensor group is 34%, then its contribution to the entire joint moment is also assumed 34% for all contractile conditions. Such an approach contains many non-trivial assumptions, among them the following:

(i) The force-length property of all knee extensor muscles has the same shape with the same optimal length (joint angle);
(ii) Antagonistic muscle activity does not contribute to the knee extensor moment;
(iii) All knee extensor muscles are activated to the same degree throughout the entire range of motion and for all (isometric, concentric, eccentric) contractile conditions;
(iv) All agonist muscles have a similar moment arm, or at least moment arms that change in proportion with the joint angle; and
(v) Relative fascicle excursions are similar across all muscles

Many of these assumptions are known to not be correct for at least some muscles that have been studied. For example, it has been shown that the joint angle of maximum moment does not necessarily coincide with the angle at which the maximum moment arm occurs [47], so, the force-length relationships of synergistic muscles are not necessarily the same [48], and submaximal activation of muscles changes fascicle optimal lengths in a complex and often unpredictable manner [49]. Finally, the optimal lengths of 2-joint muscles in a synergistic group (for example the rectus femoris in the knee extensor muscles) depend on two joint angles (hip and knee for the rectus femoris), thus contributions to moments at one joint (the knee) will depend on the configuration of the other joint (hip). Therefore, the assumption of a constant contribution of a muscle to the moment-angle relationship throughout the entire range of joint motion and at all speeds of contraction, is likely not correct. However, for lack of information, such assumptions are often made when representing human skeletal muscle function and when predicting a single muscle’s contribution to the joint moment.

Needless to say, the situation becomes infinitely more complex if we want to study muscle function during everyday movements. In such situations, not only the force-length, but also the force-velocity and history-dependent properties start playing an important role, and the muscle force is variable and transient and not at steady-state, conditions that have not been described well for single human skeletal muscles.

**Fig. 8:**
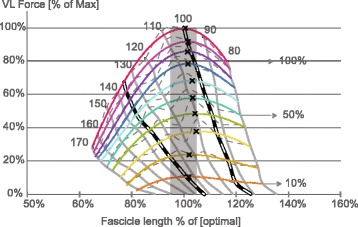
Maximal and sub-maximal force length relationship for human vastus lateralis muscle. The fascicle lengths were directly determined using ultrasound imaging while the forces were obtained making the usual assumptions discussed above. Note how the maximal and sub-maximal relationships do not scale linearly, and how optimal fascicle length, but not optimal muscle length, is about constant in this approach where the relationship was derived for sub-maximal levels of activation rather than sub-maximal levels of force. The “**x**” symbols on the graph indicate the optimal fascicle length for each of the maximal and submaximal levels of activation. The numbers on top of the graph ranging from 170 to 80 indicate the corresponding knee joint angles. (Adapted from [49], with permission)

Maybe most importantly, everyday movements are typically performed using sub-maximal levels of muscle activation. Often it is assumed that the basic muscle properties can be scaled linearly from maximal to submaximal levels of activation. However, it has been known for a long time that submaximal force-length relationships are not merely linearly scaled versions of the maximal relationship (e.g. [50, 51], and this observation, first made in isolated muscle preparations, has been reinforced recently for sub-maximal force-length relationships in human skeletal muscles [49] (Fig. 8).

#### Force-length relationships (possible solutions)

I assume that it will not be possible to measure the mechanical properties of the individual muscles comprising an agonistic group of human skeletal muscles and their respective force-time histories during everyday movements in the near future. However, theoretically at least, such measurements are relatively straight forward in an agonistic group of muscles in an animal preparation. For example, the (maximal) force-length relationships of the individual cat ankle extensor muscles have been determined [48], and the corresponding force-time histories have been determined for a variety of everyday tasks ranging from standing to walking, running, galloping, jumping, scratching and paw-shaking [52–58]. Determining the corresponding history-dependent properties, and force-velocity properties has been done partially, but submaximal relationships for these mechanical properties have not been, but could be easily determined.

#### Force-length relationships (future challenges)

Although it is fairly trivial to determine the mechanical properties of isolated muscle preparations, fibres or myofibrils, it remains a great challenge to determine the basic muscle properties for individual in vivo human skeletal muscles using voluntary (and thus inconsistent) contractions. The following challenges should be tackled in the next two decades:

(i) Develop methods for the accurate determination of in vivo human force-length (and force-velocity and history-dependent) properties for individual muscles
(ii) Develop methods for the accurate determination of these properties for submaximal and time-varying activation
(iii) Develop methods for the accurate determination of the interaction of the force-length, force-velocity and history-dependent properties for maximal steady-state and submaximal, transient (and thus functionally relevant) conditions.

#### Series elasticity (Problem)

It has been known for a long time that muscles deform during contraction. Hundreds of years ago, muscle contraction was thought to occur through the invasion of spirits that deform muscles and this deformation was thought to cause longitudinal contraction and force production. However, until approximately 30 years ago, muscle deformations were rarely acknowledged and how muscle fibre length changes differed from the length changes of entire muscles was not appreciated. The classic study by Griffith [59], who performed first fibre length measurements in a muscle of a freely moving cat, demonstrated that fibre and muscle tendon unit length changes can be in opposite directions. Griffiths [59] showed that muscle fibres shortened in the cat medial gastrocnemius at the beginning of the stance phase of walking while the muscle tendon unit was substantially stretched at that same instant in time. Since in this phase of cat walking, force is increasing, the shortening of the fascicles was associated with a corresponding stretch of the series elastic elements. Similarly, early ultrasound measurements of fascicle lengths in human skeletal muscles demonstrated that fascicles and fibres shorten as much as 20–30% in a muscle tendon unit that is contracting isometrically (i.e. the joint angle and thus the muscle tendon unit lengths were kept constant) (e.g. [60]). Again, this shortening was associated with the increase in force in isometric contractions and the corresponding stretching of serially arranged (visco-) elastic elements.

So, what is series elasticity? In a special issue of the Journal of Applied Biomechanics that was focused on the storage and release of elastic energy in skeletal muscles, the late Gerrit Jan van Ingen Schenau defined series elasticity as follows [61]:

*“the series elastic element is simply obtained by subtracting fibre length from the total muscle tendon unit length”.*

This definition has been largely accepted and used in a variety of studies in prominent journals. However, if this definition is used to make statements about the mechanics of muscles, for example to calculate the storage and release of elastic energy, then one must be careful and adhere strictly to the laws of mechanics, otherwise erroneous results may be produced and the interpretation of storage and release of elastic energy may take forms that are thermodynamically impossible.

In mechanics, the term “in series” implies that elements have the same force, or at least that the forces of in series elements are in constant proportion. For example, muscle forces are typically measured using tendon force transducers, and there is no doubt that the external tendons of muscles are in series with the muscle itself, that is, the tendon transfers the force that is produced by the muscle and the tendon force represents the muscle force.

**Fig. 9:**
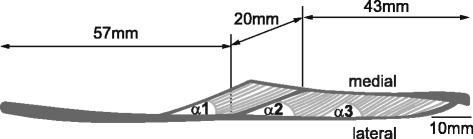
Scaled representation of a mid-longitudinal section of a cat medial gastrocnemius muscles obtained through chemical fixation. Note the pennate architecture of the muscle, the long free tendon, and the long medial and lateral aponeuroses. Using van Ingen Schenau’s definition of series elasticity (subtract fascicle length from the total muscle tendon unit length) the muscle’s series elasticity would include – and in fact be dominated – by the aponeuroses. However, since aponeuroses are clearly not in series mechanically with the tendon and/or the muscle belly, this assumption leads to erroneous results and inappropriate interpretations of the role of storage and release of elastic energy in muscle contraction (as shall be shown below)

However, if we now take a muscle, for example the medial gastrocnemius of a cat (Fig. 9) and we use the definition of series elasticity of van Ingen Schenau [61], and subtract fibre length from total muscle length, we implicitly treat the aponeuroses of the muscle as an “in series” element. However, it is easy to show that aponeuroses do not transfer the same amount of force as the tendon or the muscle, and that aponeuroses forces vary along their lengths [62]. Therefore, we must ask ourselves, what happens when one measures muscle forces (using a tendon force transducer) and then assumes that this (tendon/muscle) force is stored in a series elastic element that contains the aponeuroses, as has been done frequently in the literature?

**Fig. 10:**
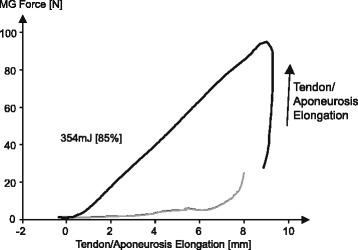
Force in the cat medial gastrocnemius as a function of changes in tendon and aponeuroses lengths obtained by subtracting fibre lengths from the total muscle tendon unit lengths. Note that plotting the muscle force against this length (defined incorrectly as the series elastic element of the muscle -[61]) results in the appearance of net work by the (incorrectly) defined series elastic element, a thermodynamic impossibility. This example illustrates that the nature of the series elastic element is difficult to define, and is often used incorrectly leading to conclusions on the storage and release of energy in muscle contraction by series elastic elements (such as aponeuroses) that are incorrect

**Fig. 11:**
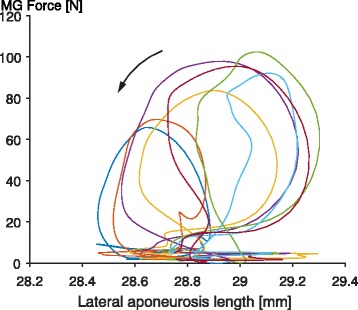
Directly measured cat medial gastrocnemius force as a function of the directly measured length of the corresponding lateral aponeuroses. Forces were measured using a standard buckle type force transducer [48, 52–59] and aponeurosis lengths were measured using two sonomicrometry crystals aligned along the mid-longitudinal collagen fascicles of the aponeurosis [83]. Note the counter-clockwise orientation of these “force-elongation” curves, and note the similar elongations of the aponeurosis in the passive muscle during the swing phase of locomotion (forces below about 10 N) and the active muscle during the stance phase of locomotion (forces between about 10 and 100 N). These direct force and elongation measurements indicate that there is no relationship between force and the elongation of the lateral aponeuroses, therefore the aponeuroses length is NOT an indicator of muscle force and is not in series with the muscle force (tendon). Furthermore, if we interpreted that the aponeurosis shown here is in series with the muscle’s contractile element or its tendon, we would obtain net work from an elastic element, an impossibility

For a typical stretch shortening cycle, starting from zero force and returning to zero force, we know that an elastic element cannot produce any net energy. In fact, a perfectly elastic element would produce zero work/energy in such a situation. However, all biological tissues, such as tendons and aponeuroses are at least slightly visco-elastic, thus there is a small loss of energy for all stretch-shortening cycles. However, if we take a muscle and calculate a “work/energy” term during locomotion by assuming that the series elastic element is obtained by subtracting the fibre/fascicle lengths from the total muscle tendon unit lengths for the entire stretch-shortening cycle and assign it the force measured at the tendon (the muscle force), then, it has been shown theoretically [62] and experimentally [45] that there is net work/energy production from the “assumed” series elastic elements, an impossibility (Fig. 10). In fact, if we measure the aponeuroses length changes in the cat medial gastrocnemius muscle directly during locomotion, and plot it against the directly measured tendon/muscle force, we obtain net work/energy from this presumed series elastic element (Fig. 11). Not only that, but Fig. 11 beautifully illustrates how the cat medial gastrocnemius aponeurosis length is essentially independent of force, and seems to behave differently when the muscle is activated (stance phase of locomotion) and when it is passive (swing phase). However, a series elastic element must elongate with increasing force and must shorten with decreasing force. Such a behavior is not observed in aponeuroses in general [45, 63, 64]. Therefore, the problem with series elasticity, when used in a mechanical context, such as storage and release of mechanical work/energy, needs to be carefully re-evaluated, and many studies have misinterpreted series elasticity, resulting in confusion and incorrect interpretation of the role of elastic elements in muscle contraction.

#### Series elasticity (solution)

The solution to the problem of series elasticity is as simple as it is relevant; only use the term series elasticity in the calculation of storage and release of mechanical energy in the mechanically correct way. Since aponeuroses are not in series with the free tendon, and thus muscle/tendon forces are not equivalent to aponeuroses forces (which vary across the length and width of the aponeuroses [62, 65], one cannot calculate the stiffness of aponeuroses or its storage and release of energy by integrating tendon force with aponeuroses deformations as is often done. Importantly, do not assume, without careful evaluation that the series elastic element of a muscle is obtained by subtracting fibre/fascicle length from the entire muscle tendon unit length, as has been suggested [61]. In most (maybe all) situations, this will lead to incorrect results, typically an overestimation of the contribution of series elastic elements to the storage and release of elastic energy in stretch-shortening cycles.

Furthermore, aponeuroses are complex 3-dimensional structures that deform based on the internal stresses of the muscles and these include pressure and shear stresses that are often not accounted for properly in muscle models [65, 66]. Also, aponeuroses do not only experience longitudinal strains but are exposed to multi-dimensional strains that may affect the longitudinal strain behavior [67, 68] and must be considered for proper understanding of aponeuroses mechanics. Finally, aponeuroses transmit variable forces along their lengths and widths [62], and these cannot be measured presently, and thus we must rely on theoretical models to predict the variable stresses in these tissues.

#### Series elasticity (future challenges)

I would love to see the following problems in whole muscle mechanics and in vivo muscle function solved:

(i) What are the true series elastic elements of muscles?
(ii) What is the exact role of the aponeuroses? What possible contributions do aponeuroses make to muscle function and muscle properties? And how can we identify the mechanical properties of aponeuroses? (note, that stiffness measurements of aponeuroses obtained from muscle force and aponeurosis length change measurements are incorrect, and estimates of aponeuroses storage and release of energy have typically been made assuming that aponeuroses transmit the same force (everywhere) as the tendon; an incorrect assumption that results (typically) in overestimates of the true storage and release of energy).
(iii) Being able to measure the true aponeuroses stresses in situ would allow for great insights into aponeuroses mechanics.

### Force-sharing among synergistic muscles

#### Force-sharing among synergistic muscles (Problems)

Arguably the most basic problem in biomechanics and movement control is the “distribution problem”. Simply formulated, the distribution problem deals with the idea of how joint moments (and thus joint movements) are accomplished by the different force carrying structures crossing a joint. The resultant joint moments, typically, can be determined easily using the so-called inverse dynamics approach [69]. For example, in order to calculate the resultant joint moments in the human lower limb during locomotion, all one needs is a force platform that measures the external ground reaction forces acting on the foot during locomotion, the three-dimensional movement of the lower limb, and the inertial characteristics (mass, moment of inertia, and centre of mass location) of the lower limb segments [69]. Once the resultant joint moments have been calculated as a function of time, it is obvious that this resultant joint moment is equipollent to the moments by all individual force carrying structures that cross the joint of interest. Structures that can contribute to the resultant joint moment are the muscles, ligaments and bony contact forces. Other structures crossing the joint (blood vessels, nerves, joint capsule, etc.) are typically assumed to not contribute to the resultant joint moment. Mathematically, the distribution problem is then expressed as:

1 $$ {M}^0=\sum_{i=1}^m\left({r}_i^m\times {f}_i^m\right)+\sum_{j=1}^l\left({r}_j^l\times {f}_j^l\right)+\sum_{k=1}^c\left({r}_k^c\times {f}_k^c\right) $$

Where *M* is the intersegmental resultant moment, and the superscript ^“0”^ designates the joint center 0; ${f}_i^m$, ${f}_j^l$, and ${f}_k^c$ are the forces in the i^th^ muscle, j^th^ ligament, and k^th^ bony contact, respectively; ${r}_i^m$, ${r}_j^l$, and ${r}_k^c$ are location vectors from the joint center to any point on the line of action of the corresponding force; “x” denotes the vector (cross) product; and m, l, and c designate the number of muscles/tendons, ligaments crossing the joints, and individual articular contact areas within the joint, respectively.

**Fig. 12:**
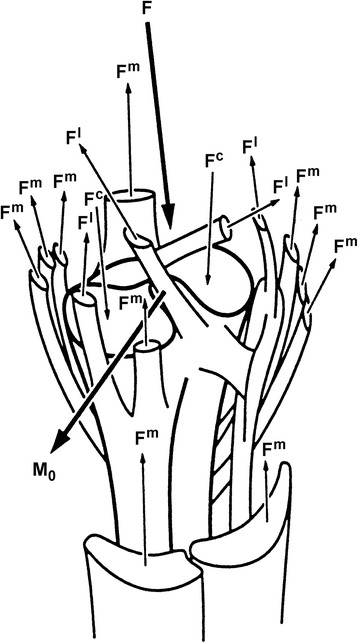
Schematic representation of the human knee with its potential force carrying structures: muscles, ligaments, and bony contacts that can contribute to the resultant inter-segmental joint forces and moments. Mathematically, this represents an indeterminate system as the resultant inter-segmental joint forces and moments represent 2 independent vector or 6 independent scalar equations with 16 force contributing elements whose force magnitude and direction result in potentially 48 unknown scalar values. Even assuming that only the muscular forces contribute substantially to the intersegmental resultant joint moment and that the direction of the muscle force vectors, and the associated moment arm vectors (direction and magnitude), are known at any instant in time, still results in a highly indeterminate system of equations with an infinite number of possible solutions for most everyday human (sub-maximal) movements. (Adapted from Crowninshield and Brand [73], with permission)

Equation (1) is captured pictorially in Fig. 12 for a human knee joint. It illustrates that the resultant knee joint moment is produced theoretically by at least 10 individual muscles, 4 individual ligaments, and 2 distinct, distributed bony contact forces. Therefore, this one-joint three-dimensional vector equation, which can be expressed as three independent scalar equations, has at least 16 unknown scalar forces (if we assume that the force vector directions for the muscle, ligament and bony contact forces are known – a non-trivial assumption). This system of eqs. (3 scalar equations with 16 independent unknown scalar forces) represents an indeterminate system, which generally, has an infinite number of solutions.

It is often assumed that within the normal range of motion, the ligament and bony contact forces contribute little if anything to the resultant intersegmental joint moment. For the knee, for example, this seems an acceptable assumption, as there is little resistance to passive knee flexion/extension within the normal range of motion. Therefore, Eq. (1) may be simplified by assuming that the muscle forces are the only contributors to the resultant joint moment; that is:

2 $$ {M}^0=\sum_{i=1}^m\left({r}_i^m\times {f}_i^m\right) $$

This vector equation can be expressed as three independent scalar equations with ten unknown muscle force magnitudes (again assuming that the muscle force direction vectors and the corresponding muscle moment arm vectors are all known – a best case scenario that contains non-trivial assumptions). Equations (1) and (2) can be solved readily using, for example, optimization theory. However the individual muscle force predictions resulting from these solutions are not accurate and are often unrealistic [54, 70–72]. But how might we approach the distribution problem in biomechanics and movement control successfully?

#### Force-sharing among synergistic muscles (possible solutions)

The force-sharing problem has been solved theoretically in a variety of ways. Static and dynamic optimization approaches have been used to solve the indeterminate mathematical system of equations using objective functions that optimize the energetics of locomotion, minimize the forces or stresses in muscles, minimize activation, and a variety of other approaches. Individual muscle forces have also been predicted using forward dynamics approaches and estimates of muscle forces based on muscle models and musculoskeletal modeling incorporating muscle activation (typically via surface electromyography, EMG) approaches (for a detailed review, of these approaches, please consult [52, 73, 74].

Experimental approaches for solving the force-sharing problem in humans do not exist to my knowledge. That is, I am not aware of studies in which multiple muscle force measurements from individual muscles of a synergistic group were measured simultaneously during normal human movement. Although there have been attempts of measuring muscle forces during human locomotion, often such measurements were performed on entire synergistic groups (for example Achilles tendon force measurements representing the triceps surae muscles - [75]), and calibration of the force measurements were typically made using “an inverse dynamics approach”, which makes it difficult to infer the absolute force values.

Shear wave elastography (SWE) has been proposed as a possible solution to identify the contributions of individual muscles to the joint moments during human movement [76]. SWE relies on the idea that the stiffness of a muscle is linearly related to the muscle force, and that the shear modulus (measured by SWE) is linearly related to the Young’s modulus. Studies in isolated in vitro muscle preparations seem to support that these two assumptions are acceptable for passively stretched muscles [77]. However, it is well known that muscle stiffness and force in active muscles are not linearly related. For example, muscles in a force enhanced state following active stretching have been found to have force as much as twice that for a purely isometric reference contraction, while stiffness of the muscle remains about the same [15]. Furthermore, changes in the shear modulus are directly related to the Young’s modulus in isotropic materials. However, muscles are not isotropic, but measurements of the shear modulus can still be related to the Young’s modulus if SWE measurements are made along the fibre direction. Small deviations from the fibre direction will result in errors of the shear modulus, Young’s modulus and force. Also, changes in the shear modulus of multiple muscles in a synergistic group have not been validated, and changes in shear modulus can presently be only expressed as corresponding changes in force, without the possibility of giving an absolute value for the force. However, with the development of this technique, or mechanically induced vibration measurements at the tendon of muscles, accurate force measurements might be possible in the not so distant future. These techniques should be explored, as techniques available for animal research, where individual muscle force measurements of synergistic muscles can be made readily [54, 55, 58, 78], remain too invasive for systematic human testing, and retain the disadvantage that proper calibration in humans is not possible.

**Fig. 13:**
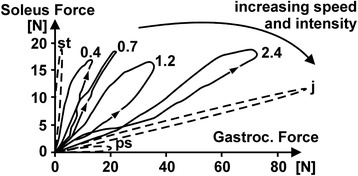
Soleus vs. medial gastrocnemius forces (Gastroc. Force) obtained by direct measurement in the cat during a variety of postural and movement tasks. Note, that variability of the force-sharing between these two muscles that occupies the entire solution space, and further note the task-specific nature of the force-sharing between these two muscles. Compare these experimentally observed results also to the common assumption that a muscle contributes force to a synergistic group in correspondence to its physiological cross-sectional area. In a cat, the physiological cross-sectional area of the soleus, and thus its maximal isometric force at optimal length, is approximately 20–25% of that of the medial gastrocnemius muscle. Nevertheless, the soleus produces substantially more force than the medial gastrocnemius for many static and dynamic tasks. (St = standing still, ps = paw shake, j = jumping (estimated from the peak forces), 0.4, 0.7 and 1.2 are the speeds of walking in m/s, 2.4 is the speed of running (trotting) at 2.4 m/s

Therefore, it appears that solution of the force-sharing problem is easiest pursued at present in animal models where multiple individual force measurements of synergistic muscles can be performed easily. Such an approach was pioneered by Walmsley [55] who measured the forces in the soleus and medial gastrocnemius muscles of freely moving cats. They found the surprising result that the small soleus (in the cat the maximal isometric soleus forces are approximately 20–25% of the maximal isometric medial gastrocnemius forces) contributed more force to normal walking and slow trotting than the much bigger medial gastrocnemius muscle. We extended that approach to measure as many as four muscle forces simultaneously in the cat hind-limb muscles and solving the force-sharing problem theoretically, thus allowing for comparison of the experimentally measured and theoretically calculated individual muscle forces [54, 71, 72]. However, even with such an approach, it has been impossible to develop an algorithm that predicts individual muscle forces as a function of time accurately (where I define accurate as within ±5% of the measured value at all times). In fact, it seems virtually impossible to predict the wide variety of force-sharing observed experimentally in muscles, such as that between the cat soleus and medial gastrocnemius muscles, where it is possible to have substantial force in the soleus and no force in the medial gastrocnemius (standing still), have substantial medial gastrocnemius and no soleus forces (scratching and paw-shaking), and anything in between these two extremes for locomotion, jumping and climbing movements (Fig. 13).

**Fig. 14:**
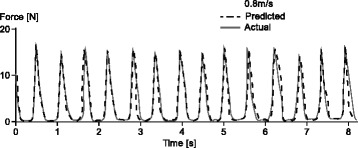
Illustration of the prediction of individual muscle forces using an artificial neural network (ANN) approach. In this example, the directly measured soleus forces (solid trace) in a freely moving cat are predicted (dashed trace) solely based on EMG patterns during walking. The ANN was trained with input of soleus force and EMG obtained from another cat. The force predictions are among the best dynamic and submaximal force predictions ever published but they provide little insight into how these forces are controlled and how they are achieved

Musculoskeletal modeling in conjunction with EMG driven muscle models have been used frequently to predict individual muscle forces in human movement, but appropriate validation has been lacking, and thus these attempts must be considered with caution. Again, using animal models in which EMG and muscle forces are measured directly offer unique possibilities for developing and validating EMG driven muscle models. Artificial Neural Network, Adaptive Filtering and many other pattern recognition tools have proven powerful in predicting dynamic individual muscle forces accurately and reliably [79–81] (Fig. 14). However, these approaches invariably require that the pattern recognition software (for example the artificial neural network) is trained with experimental data, thus individual and calibrated muscle force measurements must be made at one point, and this seems virtually impossible for human movements with the currently available technology. Furthermore, although the individual muscle force predictions using artificial neural network approaches have been shown to be impressive, these numerical approaches provide little (if any) insight into the relationship between the mechanics of the muscle, its properties and activation, and the corresponding resultant force. As such, these force predictions could be valuable from an engineering point of view if knowledge of muscle forces are the ultimate goal, but are disappointing from a scientific point of view when attempting to understand how individual muscle forces are controlled in a synergistic group and how these forces are produced.

#### Force-sharing among synergistic muscles (future challenges)

The force-sharing, or redundancy problem, in biomechanics and movement control has been recognized and described for more than half a century (e.g. [82]). Despite the fundamental importance of this problem, and despite great scientific efforts, we are still not able to predict individual muscle forces accurately during human movement and have no accurate, non-invasive, and simple way of measuring individual muscle forces experimentally during human movement. And although I could list a great number of challenges for future research in this area, in one way or another, they can all be summarized under two big topics: the first of these topics is more fundamental, the second more applied and technical.

The first (and fundamental) problem that needs solution in the future is an understanding of how animals, including humans, recruit muscles and how they use them in everyday movements. This challenge requires a series of sub-challenges to be solved: for example, we need to understand how the nervous system activates muscles in detail, what the properties of the muscles are that translate the activation into muscle force, and how this muscular coordination operates for all the different movements we can produce.

The second (and more applied) challenge will be to develop a method that allows for simple, non-invasive, and accurate measurement of individual muscle forces in animals, including humans. I believe that this problem can and will be solved over the next twenty years and will catapult our understanding of animal movements and locomotion into new and exciting dimensions.

## Conclusions

Looking ahead to the next BANCOM meeting in 20 years from now (i.e., in 2036), I hope that the following problems and questions will have been solved in the three areas I discussed here. First, we will understand the mechanics of eccentric contractions in skeletal muscles much better than we do now. Specifically, I anticipate that the molecular details and functions of titin (and possibly other structural proteins) in eccentric contractions are fully elucidated. Second, we will know the mechanical properties and the functions of individual muscles for sub-maximal, dynamic conditions as occur in everyday human movements, and third, we will be able to quantify individual muscle forces in human movements reliably and accurately and will have solved the distribution problem in biomechanics and movement control.
