# Correction to: skeletal muscle mechanics: questions, problems and possible solutions

**DOI:** 10.1186/s12984-018-0351-5

**Published:** 2018-03-07

**Authors:** W. Herzog

**Affiliations:** Faculty of Kinesiology, University of Calgary, 2500 University Dr, Calgary, AB T2N-1N4 Canada

## Correction

In Fig. 3a of the original manuscript [1], the passive force at the beginning of the force-time history (approximately from 0 to 1 s) of each of the black and orange traces was interchanged in the coloring process. This was an error in the artwork preparation, not the original data. The corrected figure is shown below.

**Fig. 3:**
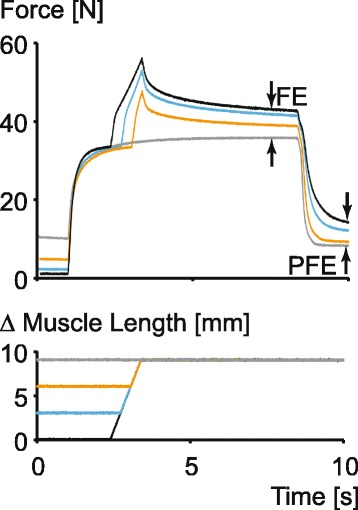
Force enhancement property of skeletal muscle as experimentally observed in a whole, intact muscle (**a**) and in a single, mechanically isolated sarcomere (**b**). Note that the steady-state isometric force following an active stretch is substantially greater than the corresponding steady-state force for a purely isometric reference contraction at the same length and with the same amount of activation (indicated as FE in both figures). Furthermore, the force enhancement often also contains a passive component, indicated by PFE in fig. **a** Note also, the increase in force above that observed at optimal sarcomere length following active stretching of a single sarcomere (O-FE in Fig. **b**). Finally, note that the amount of force enhancement is increased with increasing stretch magnitude (in Fig. **a**)

I would like to thank Brent Raiteri for alerting me to this error.
